# DSDAN: A Dual-Stream Multimodal Log-Flow Fusion Framework for Network Configuration Auditing and Compliance-Risk Detection

**DOI:** 10.1371/journal.pone.0357204

**Published:** 2026-09-08

**Authors:** Mohammed Saad Javeed, MD Al Rafi, Arifur Rahman, Hashibul Ahsan Shoaib, Sheikh Razia Sultana

**Affiliations:** 1 Information Science, Trine University, Allen Park, Michigan, United States of America; 2 Washington University of Science and Technology, Eisenhower Ave, Alexandria, Virginia, United States of America; 3 School of Business, International American University, Los Angeles, California, United States of America; 4 Information Technology, St. Francis College, Brooklyn, NewYork, United States of America; 5 Jahangirnagar University, Savar, Dhaka, Bangladesh; PLOS: Public Library of Science, UNITED KINGDOM OF GREAT BRITAIN AND NORTHERN IRELAND

## Abstract

The increasing complexity of modern networks, particularly in IoT and distributed cloud environments, poses significant challenges for maintaining configuration integrity and compliance. Existing solutions for network auditing rely heavily on static rules or manual scripting, which fail to scale or adapt to dynamic network conditions. In this work, we propose a novel AI-driven framework, the Dual-Stream Deep Auditing Network (DSDAN), that leverages deep learning to automate configuration auditing and detect policy violations. DSDAN integrates structured network flow features and unstructured device logs through parallel encoder-decoder streams, enabling joint representation learning for robust compliance analysis. For evaluation, we combine IoT Device Network Logs and UNSW-NB15 because they represent two complementary evidence channels used in practical network auditing: device-level operational logs and flow-level behavioral security records. IoT logs support reconstruction-based identification of abnormal device or configuration behavior, while UNSW-NB15 provides labeled network-flow patterns for modeling unauthorized, anomalous, and attack-like activity. Using these complementary sources, DSDAN achieves an overall accuracy of 93.2%, macro F1-score of 0.918, and micro F1-score of 0.927, surpassing baseline models. The model further records an AUC of 0.957, PR-AUC of 0.948, and the lowest IoT log reconstruction error (MSE = 0.031, MAE = 0.020). Despite its dual-stream architecture, DSDAN maintains efficient inference with a latency of 3.1 ms and memory footprint of 110 MB. These results validate the effectiveness of our approach in identifying subtle misconfigurations and unauthorized behaviors often missed by traditional tools.

## 1. Introduction

The rapid proliferation of interconnected devices, driven by the growth of the Internet of Things (IoT), cloud computing, and software-defined infrastructure, has made modern networks more dynamic and distributed than ever before [1,2]. These systems enable high flexibility and automation but also introduce substantial complexity in configuration management and policy enforcement [3,4]. Misconfigurations, outdated scripts, and inconsistent compliance practices are now major contributors to security incidents, often more so than external attacks [5].

While significant advances have been made in intrusion detection and anomaly detection using machine learning, comparatively less attention has been paid to automating network configuration auditing and compliance verification [6,7]. Most existing tools rely on static rule sets or manual scripting in languages like Python or Perl, which are brittle and require constant maintenance. Furthermore, these approaches often overlook subtle violations, especially those emerging in heterogeneous and rapidly evolving environments like industrial IoT or multi-tenant cloud systems [8,9].

This work addresses that gap by proposing a unified deep learning framework for AI-driven network configuration auditing. Our goal is to develop a data-driven system that can automatically detect misconfigurations, policy violations, and anomalous behavior by learning from both structured flow-level data and unstructured network logs. Specifically, we introduce a novel architecture, Dual-Stream Deep Auditing Network, that integrates heterogeneous network data into a unified detection pipeline. The motivation stems from the operational need for adaptable, low-maintenance auditing systems that can respond to changing network behavior without manual rule tuning.

The proposed methodology employs parallel encoder-decoder streams for each data modality, followed by a joint feature fusion module and supervised classification layers. This design enables the model to capture both low-level anomalies and high-level compliance deviations without the need for handcrafted rules or predefined templates. The significance of this research lies in its shift from rule-based compliance checks to intelligent, model-driven auditing. This work not only enhances detection capabilities but also reduces the dependence on human experts and scripted logic. Our contributions can be summarized as follows:

- We propose DSDAN, an auditing-oriented dual-stream multimodal framework that adapts standard representation learning and fusion components to the specific task of policy-aligned compliance-risk detection through joint modeling of IoT log deviations and flow-level behavioral evidence.
- We develop a preprocessing and training pipeline capable of handling real-world datasets from diverse sources, including IoT device logs and synthetic attack traffic.
- We demonstrate, through extensive experiments, that our model significantly outperforms state-of-the-art methods across multiple evaluation metrics, including accuracy, F1-score, and AUC.

The remainder of this paper is organized as follows. Section 2 presents the related works. Section 3 introduces the proposed model architecture and training procedure. Section 4 presents experimental results and evaluation. Section 5 discusses implications, limitations, and future directions. Finally, Section 6 concludes the paper.

## 2. Related work

Network security and auditing have been long-standing challenges in the field of computer and communication systems, particularly as networks become more heterogeneous, software-defined, and dynamically reconfigurable [10]. Prior research in this space can be broadly classified into three major categories: rule-based compliance systems, classical machine learning approaches, and modern deep learning models. While each category has contributed meaningfully to improving network monitoring and threat detection [11], several limitations persist when these methods are applied to real-time configuration auditing and compliance verification in evolving environments such as IoT deployments, cloud-native architectures, and enterprise-grade hybrid infrastructures [12].

Traditional rule-based compliance and auditing mechanisms rely heavily on static logic encoded using SNMP traps, syslog analyzers, regular expressions, and configuration templates. These systems, typically implemented through custom scripts in Python, Perl, or shell environments, require human-defined rules to detect known policy violations or misconfigurations. While effective in stable environments, these systems do not scale well with changes in network topology or device diversity [13]. More critically, they cannot detect unknown or emergent anomalies and often suffer from high false negative rates in previously unseen scenarios. The lack of adaptability and high maintenance burden limit their application in large-scale deployments with high configuration churn.

To overcome the rigidity of rule-based systems, classical machine learning algorithms have been applied to structured network telemetry data such as NetFlow, sFlow, and packet metadata. Models like decision trees, random forests, support vector machines (SVM), and Naive Bayes classifiers have shown utility in detecting well-characterized anomalies, including DoS, port scans, and protocol misuse [14]. However, these models depend on carefully engineered features, suffer from limited generalization to heterogeneous device types, and are typically trained on balanced datasets that do not reflect real-world class imbalances [15]. Recent IDS studies have also explored pattern matching, encoded feature representation, and feature selection to improve detection efficiency. Rashid et al. examined matching algorithms for IDS based on DNA encoding, showing how symbolic sequence representation can support attack identification in signature-oriented detection pipelines [16]. Related work further extended this direction through DNA-encoding-based feature selection, where compact selected features were used to reduce computational cost while preserving intrusion detection performance [17]. In addition, four-character DNA encoding has been applied to anomaly IDS design, demonstrating the value of compact symbolic representation for network attack detection [18]. These studies strengthen the broader IDS literature; however, they mainly focus on matching efficiency, encoded representation, and feature selection. They do not directly address multimodal log-flow fusion for configuration auditing, where unstructured device logs and structured flow records are jointly modeled to identify configuration drift, policy violations, behavioral anomalies, and confirmed attacks.

Recent work on network digital twins, configuration repositories, and graph-based compliance systems further highlights the need to connect operational network evidence with policy-oriented reasoning. Network digital twin research emphasizes virtual network replicas and data-driven models for scenario analysis, change assessment, and real-time operational insight [19]. Configuration-verification studies such as FastCUV use flow models and graph structures to verify the impact of network configuration updates [20]. CMDB-oriented studies show that centralized configuration repositories can support cloud-resource inventory, change tracking, impact analysis, and security compliance management [21]. Knowledge-graph-based compliance systems represent infrastructure and policies through formal semantics and automated reasoning, allowing non-compliant services to be identified from graph-structured configuration knowledge [22]. In parallel, recent multimodal fusion surveys emphasize the importance of robust integration under noisy, incomplete, or quality-varying modalities [23]. These directions differ from DSDAN because they focus mainly on model-based verification, configuration repositories, semantic rule reasoning, or general fusion reliability, whereas DSDAN studies deep log-flow evidence fusion for policy-aligned compliance-risk detection using complementary audit-evidence sources.

The recent emergence of deep learning has brought powerful tools to the field of network intrusion detection and behavioral analytics. Convolutional neural networks (CNNs) have been applied to network traffic as spatial data, while recurrent architectures like LSTM, GRU, and BiLSTM are used to model sequential patterns in event logs and packet traces [24,25]. Autoencoders, variational autoencoders, and hybrid combinations with LSTM decoders have further enabled unsupervised and semi-supervised detection of anomalies in high-dimensional data [26,27]. Nevertheless, most deep learning solutions focus on attack detection in packet-level data and do not explicitly address configuration violations, policy compliance checks, or behavioral inconsistencies in logs. Their application is often confined to benchmark datasets and lacks the real-time context needed for compliance auditing [28].

In IoT-specific environments, where devices emit both structured telemetry and unstructured logs, a few hybrid approaches have attempted to combine these modalities. Existing solutions often use manual feature fusion or parallel pipelines without joint optimization (e.g., hybrid CNN‑LSTM with feature concatenation for IoT traffic analysis) [29] or aligned multimodal fusion of packet headers and payloads without log integration [30]. As a result, they suffer from feature misalignment, loss of semantic context, and suboptimal learning of interdependencies between flow-level and behavioral signals. Furthermore, these methods usually remain focused on threat detection (e.g., botnets, malware) rather than configuration verification or auditing, which involves a different problem space and evaluation criteria.

Across all these categories, several critical gaps remain. First, most existing models assume static behavior, while real-world networks evolve rapidly with changing roles, configurations, and access policies [31]. Second, few models are explicitly designed to detect compliance violations, deviations from prescribed configuration baselines, rather than direct attacks [32]. Third, many methods are single-modal, unable to synthesize structured flow data with unstructured logs that carry rich semantic information about system and user behavior. Finally, explainability and low-latency inference, both crucial for real-world deployment, are often neglected in favor of raw detection performance [33].

To address these limitations, this paper proposes the *Dual-Stream Deep Auditing Network (DSDAN)*, a novel architecture tailored for real-time and retrospective network configuration auditing. Unlike traditional intrusion detection systems, DSDAN is explicitly designed to capture compliance violations and configuration drift by learning from both structured network features and unstructured IoT device logs. Its parallel encoding streams allow independent modeling of different modalities, while the fusion module enables context-aware decision-making. This approach overcomes the brittleness of rules, the rigidity of classical models, and the narrow scope of existing deep learning solutions. The novelty of DSDAN is not limited to the use of multimodal fusion itself, since feature concatenation, parallel encoders, and autoencoder-based representation learning are established techniques in deep learning. The main distinction lies in how these components are adapted to policy-aligned compliance-risk detection. The IDS and multimodal anomaly detection studies discussed above mainly focus on matching efficiency, encoded representation, feature selection, traffic classification, or generic anomaly detection. In contrast, DSDAN combines an unsupervised IoT log reconstruction stream with a supervised flow-sequence classification stream to jointly model log-level deviation evidence and flow-level behavioral evidence. The fused representation is then mapped to auditing-oriented compliance-risk categories, including policy drift, configuration violation, behavioral anomaly, and confirmed attack. Thus, the contribution lies in the task-specific formulation and validation of log-flow fusion for compliance-risk auditing, where heterogeneous log-derived and flow-derived evidence are jointly optimized for audit-relevant risk prediction.

## 3. Methodology

This section outlines the architecture and learning framework of the proposed Dual-Stream Deep Auditing Network (DSDAN), designed to detect network configuration anomalies and behavioral policy violations. The method integrates unsupervised representation learning from unstructured IoT logs and supervised classification from structured flow-level behavioral security records.

### 3.1. Data preprocessing

To ensure the effectiveness and generalizability of our proposed Dual-Stream Deep Auditing Network, we applied a multi-stage data preprocessing pipeline tailored separately for the IoT device logs and the UNSW-NB15 dataset. The goal was to prepare clean, structured, and semantically rich data suitable for deep learning architectures with attention to consistency, normalization, and representation learning. We adopted a combination of classical techniques and modern deep learning-oriented strategies, including feature engineering, token embedding, and unsupervised representation learning.

#### 3.1.1. IoT device network logs.

The IoT logs, recorded in semi-structured CSV format, contain fields such as timestamps, source and destination IP addresses, port numbers, message payloads, and device metadata. Since this dataset is unlabeled and primarily log-based, we processed it as a sequence of structured log entries.

1. Log Parsing and Tokenization: Each log entry $l_{i}$ was parsed into a sequence of discrete fields and payload tokens:

$$\begin{array}{c} l_{i}=\{t_{i},s_{i},d_{i},p_{i},m_{i}\} \end{array}$$ (1)

where $t_{i}$ is the timestamp, $s_{i}$ and $d_{i}$ are source and destination IPs, $p_{i}$ is the port, and $m_{i}$ is the message payload. The payload $m_{i}$ was tokenized using byte-pair encoding (BPE) for subword-level generalization.

2. Timestamp Normalization: We normalized timestamps using min-max scaling:

$$\begin{array}{c} {\hat{t}}_{i}=\frac{t_{i}-\min(T)}{\max(T)-\min(T)} \end{array}$$ (2)

where *T* is the set of all timestamps. This scaling preserved temporal relationships and made the time dimension compatible with neural network inputs.

3. IP Address Encoding: IPs were numerically encoded via learned embeddings. Each unique IP address x∈𝒳 was mapped using:

$$\begin{array}{c} {𝐞}_{x}=\text{Embed}(x)\in {\mathbb{R}}^{d} \end{array}$$ (3)

This representation captures similarity across devices and flows based on learned traffic behavior.

4. Autoencoder-Based Feature Learning: To obtain unsupervised latent representations from the preprocessed IoT logs, we trained a sequence autoencoder:

$$\begin{array}{c} {𝐳}_{i}=f_{\theta }(l_{i}),\;{\hat{l}}_{i}=g_{\phi }({𝐳}_{i}) \end{array}$$ (4)

where $f_{\theta }$ is the encoder, $g_{\phi }$ is the decoder, and ${𝐳}_{i}$ is the learned latent vector. We minimized reconstruction loss:


$$\begin{array}{c} {ℒ}_{\text{rec}}=\sum_{i}\|l_{i}-{\hat{l}}_{i}{\|}^{2} \end{array}$$ (5)

#### 3.1.2. UNSW-NB15 dataset.

The UNSW-NB15 dataset consists of labeled network flow records with 49 features and 10 class labels (normal + 9 attack types). This dataset was used for supervised training of the behavioral auditing branch.

1. Feature Selection and Cleaning: We removed redundant identifiers (e.g., id, label_name) and retained only numeric and categorical fields relevant for traffic analysis. Missing values were filled using median imputation:

$$\begin{array}{c} x_{i}^{\left(j\right)}=\left\{ \begin{array}{cc} x_{i}^{\left(j\right)} & \text{if }x_{i}^{\left(j\right)}\neq \text{NaN}\\ \text{Median}(x^{\left(j\right)}) & \text{otherwise} \end{array}\right. \end{array}$$ (6)

2. Categorical Encoding: Protocol and service fields were one-hot encoded:

$$\begin{array}{c} {𝐯}_{i}=\text{OneHot}({\text{proto}}_{i},{\text{service}}_{i}) \end{array}$$ (7)

3. Feature Normalization: All continuous features were standardized using z-score normalization:

$$\begin{array}{c} x_{i}^{\left(j\right)}=\frac{x_{i}^{\left(j\right)}-\mu_{j}}{\sigma_{j}} \end{array}$$ (8)

where $\mu_{j}$ and $\sigma_{j}$ are the mean and standard deviation of feature *j*.

4. Sequence Construction: To model temporal dependencies in traffic behavior, we grouped flows into sequences of *k* = 10 using sliding windows:

$$\begin{array}{c} S_{i}=\{x_{i},x_{i+1},...,x_{i+9}\},\;y_{i}^{S}=\left\{ \begin{array}{cc} y_{i}, & \text{if all labels in }S_{i}\text{ are identical},\\ \text{arg}\max_{y\in \{y_{i},...,y_{i+9}\}}r(y), & \text{otherwise}, \end{array}\right. \end{array}$$ (9)

where *r*(*y*) denotes the risk priority assigned to each class. This risk-preserving rule avoids suppressing minority or high-risk attack labels in mixed windows, unlike a simple majority-vote rule. For the reported experiments, sequence windows were generated with a non-overlapping stride of *k* = 10 to avoid overlap-induced leakage between adjacent flow sequences. Thus, the official UNSW-NB15 test partition containing 82,332 flow records produced 8,233 test sequences after discarding the final two incomplete records. To verify that windowing did not remove minority classes, we compared the class distribution before and after sequence construction. At the record level, the test partition contained Normal: 37,000, Fuzzers: 6,062, Analysis: 677, Backdoors: 583, DoS: 4,089, Exploits: 11,132, Generic: 18,871, Reconnaissance: 3,496, Shellcode: 378, and Worms: 44 records. After non-overlapping windowing and risk-preserving label assignment, the corresponding sequence-level counts were Normal: 3,698, Fuzzers: 606, Analysis: 68, Backdoors: 58, DoS: 409, Exploits: 1,113, Generic: 1,887, Reconnaissance: 350, Shellcode: 38, and Worms: 6. These counts show that minority attack categories remain represented after windowing, while the final sequence-level evaluation uses all 8,233 test sequences.

#### 3.1.3. Dataset partitioning.

After preprocessing, we partitioned both datasets into training, validation, and test sets with the following ratios:


$$\begin{array}{c} \text{Train}:\text{Validation}:\text{Test}=70\%:15\%:15\% \end{array}$$ (10)

We ensured temporal and class distribution consistency by applying stratified sampling for labeled data and uniform time-block sampling for the IoT logs.

#### 3.1.4. Final tensor construction.

The final input tensors for the dual-stream model were constructed as:


$$\begin{array}{c} \begin{array}{ccc} {𝒳}_{\text{IoT}} & = & \{{𝐳}_{1},...,{𝐳}_{n}\}\in {\mathbb{R}}^{n\times d_{z}} \end{array} \end{array}$$ (11)

$$\begin{array}{c} \begin{array}{ccc} {𝒳}_{\text{UNSW}} & = & \{S_{1},...,S_{m}\}\in {\mathbb{R}}^{m\times k\times d_{f}} \end{array} \end{array}$$ (12)

where $d_{z}$ is the latent dimension from the IoT autoencoder, *k* is the sequence length, and $d_{f}$ is the number of features per flow record.

This structured preprocessing enables our deep auditing architecture to efficiently learn from both configuration-level log sequences and flow-level behavioral patterns, supporting robust compliance anomaly detection in hybrid network environments.

The IoT log dataset and UNSW-NB15 flow dataset are treated as complementary audit-evidence sources. The IoT stream captures device-level operational and configuration-related behavior through reconstruction-based log representation learning, while the UNSW-NB15 stream captures flow-level communication and security behavior through supervised sequence modeling. The proposed framework aligns these sources after modality-specific encoding, where both streams are projected into a common latent audit-evidence space. This evidence-level alignment is appropriate for network auditing because operational logs and flow records often provide different but complementary views of the same broader security and compliance context. The fusion stage therefore evaluates how device-level log deviations and flow-level behavioral patterns jointly contribute to compliance-risk detection.

### 3.2. Proposed methodology

This section presents the DSDAN framework, designed to jointly detect static misconfigurations in IoT logs and dynamic anomalies in network traffic through a dual-branch learning pipeline.

#### 3.2.1. Problem definition.

Given two complementary audit-evidence sources, we formulate the task as multimodal compliance-risk detection from device-level operational logs and flow-level behavioral security records:

- $D_{IoT}=\{l_{1},l_{2},...,l_{n}\}$: IoT device log entries used to learn normal operational and configuration-related log structure through reconstruction-based representation learning.
- $D_{UNSW}=\{(x_{1},y_{1}),(x_{2},y_{2}),...,(x_{m},y_{m})\}$: labeled network flow sequences used to learn behavioral security patterns associated with normal, anomalous, unauthorized, and attack-like traffic.

This formulation follows the practical observation that network auditing is rarely based on a single homogeneous data source. Auditors and monitoring systems commonly interpret device logs and network flows together because logs describe device-level operational behavior, while flow records describe communication behavior and security-relevant activity. In DSDAN, low log reconstruction error and normal flow behavior support the compliant category. Increasing reconstruction deviation indicates abnormal operational or configuration behavior and is used to identify policy drift or configuration-violation risk. Flow-level attack or anomaly labels provide behavioral evidence for behavioral anomaly and confirmed attack categories.

The five output categories are therefore defined as audit-relevant compliance-risk abstractions. They consolidate the original UNSW-NB15 flow taxonomy according to auditing severity and behavioral meaning while incorporating reconstruction-based evidence from the IoT log stream. The normal UNSW-NB15 class supports compliant behavior, lower-severity or probing traffic patterns such as Fuzzers, Analysis, and Reconnaissance support behavioral-anomaly evidence, and higher-risk attack categories such as Backdoors, DoS, Exploits, Generic, Shellcode, and Worms support confirmed-attack evidence. The IoT log stream complements these flow labels by contributing reconstruction-based evidence of abnormal device or configuration behavior. This design allows the model to learn a unified risk representation from heterogeneous but audit-relevant evidence sources.

Our goal is to learn a unified model $F_{\theta }$ that detects:

- Log-level deviation patterns that indicate policy drift or possible configuration violation.
- Flow-level behavioral anomalies and attack patterns that indicate elevated compliance risk.
- A fused compliance-risk category derived from both log and flow evidence.

#### 3.2.2. Model pipeline.

The DSDAN consists of two parallel branches:

1. Unsupervised IoT Stream: Learns latent configuration states from IoT logs using a sequence autoencoder.
2. Supervised Flow Stream: Classifies traffic sequences into compliance classes using a CNN-BiLSTM model.

Both branches share representations at an intermediate fusion layer and output a compliance risk score.

#### 3.2.3. IoT log representation learning.

Each preprocessed IoT log sequence $l_{i}$ is passed through an encoder network $f_{\theta }$:


$$\begin{array}{c} {𝐳}_{i}=f_{\theta }(l_{i})\in {\mathbb{R}}^{d_{z}} \end{array}$$ (13)

where ${𝐳}_{i}$ is the latent configuration representation. The reconstruction is computed by a decoder $g_{\phi }$:


$$\begin{array}{c} {\hat{l}}_{i}=g_{\phi }({𝐳}_{i}) \end{array}$$ (14)

We minimize the reconstruction loss:


$$\begin{array}{c} {ℒ}_{\text{IoT}}=\sum_{i=1}^{n}\|l_{i}-{\hat{l}}_{i}{\|}_{2}^{2} \end{array}$$ (15)

Latent vectors ${𝐳}_{i}$ are then passed to the fusion layer for compliance reasoning.

#### 3.2.4. Flow-based supervised classification.

Let $x_{i}=\{x_{i}^{\left(1\right)},x_{i}^{\left(2\right)},...,x_{i}^{\left(k\right)}\}$ be a sequence of *k* flows. Each $x_{i}^{\left(j\right)}\in {\mathbb{R}}^{d_{f}}$ is a feature vector. We first apply a CNN to extract local flow patterns:


$$\begin{array}{c} {𝐡}_{i}^{\left(j\right)}=\text{CNN}(x_{i}^{\left(j\right)}) \end{array}$$ (16)

The CNN outputs are passed to a BiLSTM to capture temporal dependencies:


$$\begin{array}{c} {𝐬}_{i}=\text{BiLSTM}(\{{𝐡}_{i}^{\left(1\right)},...,{𝐡}_{i}^{\left(k\right)}\})\in {\mathbb{R}}^{2h} \end{array}$$ (17)

A softmax classifier maps the sequence state ${𝐬}_{i}$ to a predicted compliance label:


$$\begin{array}{c} {\hat{y}}_{i}=\text{arg}\max\text{Softmax}(W{𝐬}_{i}+b) \end{array}$$ (18)

The classification loss is defined using cross-entropy:


$$\begin{array}{c} {ℒ}_{\text{flow}}=-\sum_{i=1}^{m}\sum_{c=1}^{C}𝕀(y_{i}=c)\log P(y_{i}=c|x_{i}) \end{array}$$ (19)

The CNN-BiLSTM design was selected because the flow stream uses short sequential windows of network records, where local packet-flow interactions and bidirectional temporal dependencies are both relevant. The convolutional layer captures local feature transitions among adjacent flow records, while the BiLSTM models forward and backward temporal context within each window. Transformer-based encoders are powerful for long-range sequence modeling, but their self-attention mechanism introduces higher computational cost and typically benefits from larger training corpora or longer sequences. Since the auditing pipeline targets low-latency compliance detection with compact flow windows, CNN-BiLSTM provides a suitable balance between temporal modeling capacity, parameter efficiency, and inference speed.

#### 3.2.5. Fusion and joint reasoning.

To jointly reason over static and dynamic perspectives, we fuse the representations:


$$\begin{array}{c} {𝐟}_{i}=\text{Fusion}([{𝐳}_{i}\;\|\;{𝐬}_{i}])\in {\mathbb{R}}^{d_{f}} \end{array}$$ (20)

Here, [·‖·] denotes vector concatenation after both modality-specific representations are projected into a common latent audit-evidence space. The index *i* denotes the training-batch instance used for joint optimization of the two evidence streams, not a raw timestamp-level or packet-level record match. This design is based on evidence-level fusion: the IoT branch represents log-level operational and configuration-deviation evidence, while the flow branch represents traffic-level behavioral and security evidence. By aligning both representations in the latent space, the model learns whether the combination of log-derived and flow-derived evidence improves compliance-risk prediction. The fused vector is passed through fully connected layers:


$$\begin{array}{c} {\hat{y}}_{i}=\text{Softmax}(\text{ReLU}(W_{2}\cdot \text{ReLU}(W_{1}{𝐟}_{i}+b_{1})+b_{2})) \end{array}$$ (21)

**Fig 1:**
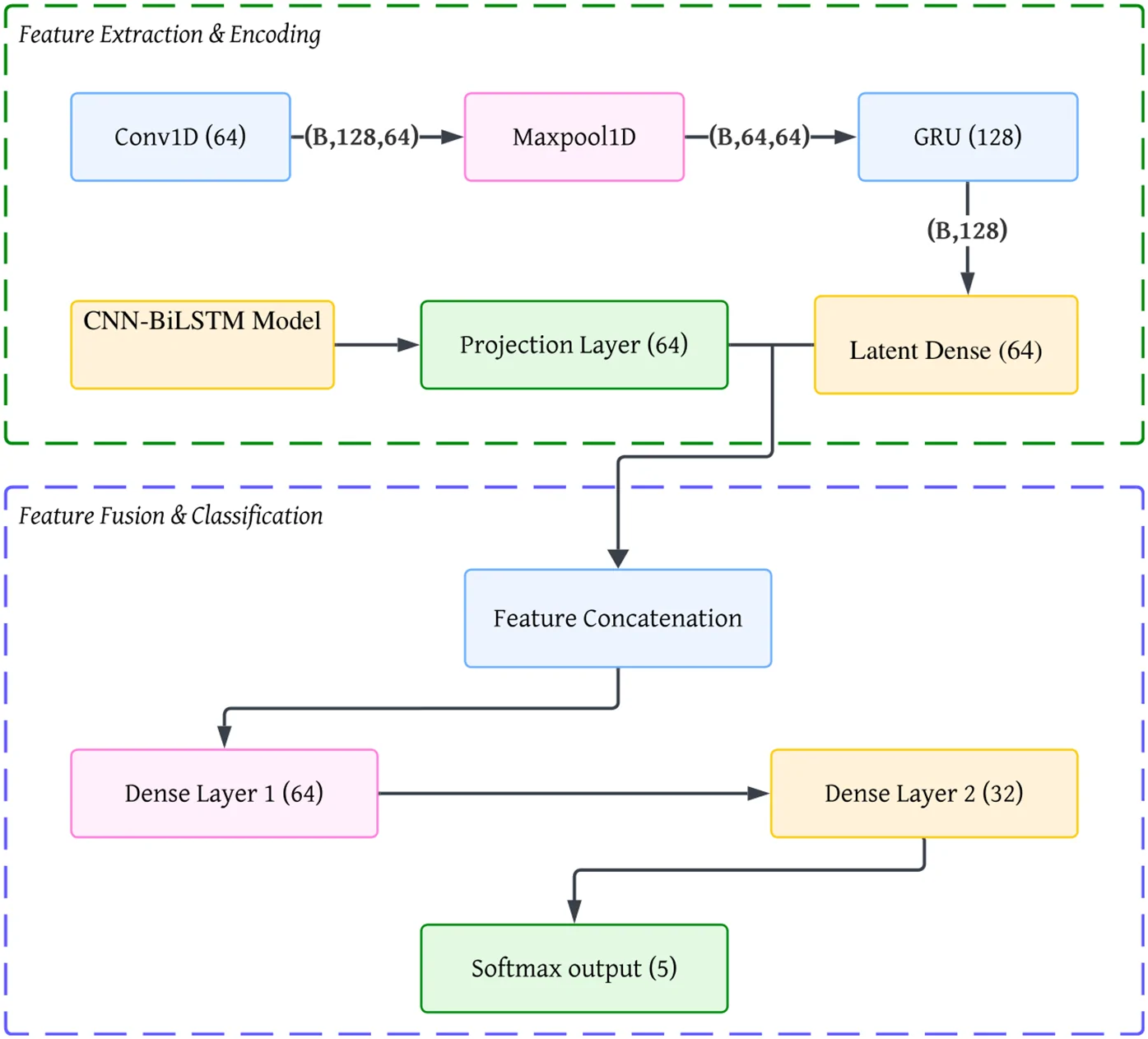
Detailed layer-wise pipeline of the DSDAN model.

The fusion module provides task-specific evidence integration for auditing-oriented risk prediction. The IoT stream contributes reconstruction-based deviation evidence from device logs, while the flow stream contributes supervised behavioral evidence from network traffic sequences. Combining these representations allows the classifier to distinguish cases where a flow-level anomaly is supported by log-level deviation from cases where only one modality shows abnormal behavior. Thus, the dual-stream design is defined by its compliance-risk formulation, joint optimization of heterogeneous evidence sources, and empirical validation through ablation. A detailed layer-wise pipeline of the DSDAN model is shown in Fig 1.

### 3.3. Architectural details

The Dual-Stream Deep Auditing Network is composed of two branches: (i) an unsupervised autoencoder for IoT log representation, and (ii) a supervised CNN-BiLSTM classifier for flow-level behavioral security modeling. Both branches converge at a fusion layer for final compliance-risk classification. Table 1 provides a detailed layer-by-layer specification of the complete architecture.

**Table 1:** Layer-wise architectural specifications of DSDAN.

Component	Layer Type	Output Shape	# Params	Activation
**IoT Autoencoder Branch**				
Input (Tokenized Log)	–	(*B*, 128, 64)	–	–
Encoder Conv1D	Conv1D (64)	(*B*, 128, 64)	12,352	ReLU
Encoder MaxPooling	MaxPool1D	(*B*, 64, 64)	0	–
Encoder GRU	GRU (128)	(*B*, 128)	74,496	tanh
Latent Dense Layer	Dense (64)	(*B*, 64)	8,256	ReLU
Decoder Dense Layer	Dense (128)	(*B*, 128)	8,320	ReLU
Decoder Repeat Vector	RepeatVector(64)	(*B*, 64, 128)	0	–
Decoder GRU	GRU (64)	(*B*, 64, 64)	37,248	tanh
Decoder TimeDistributed	Dense (64)	(*B*, 64, 64)	4,160	Sigmoid
**UNSW Flow Branch**				
Input (Flow Sequence)	–	(*B*, 10, 49)	–	–
1D Convolution	Conv1D (64)	(*B*, 10, 64)	10,112	ReLU
BatchNorm + Dropout	BN + Dropout(0.3)	(*B*, 10, 64)	256	–
BiLSTM	BiLSTM (128)	(*B*, 256)	198,656	tanh
Dense Projection Layer	Dense (64)	(*B*, 64)	16,448	ReLU
**Fusion and Classifier**				
Fusion Concatenation	–	(*B*, 128)	–	–
Dense Layer 1	Dense (64)	(*B*, 64)	8,256	ReLU
Dense Layer 2	Dense (32)	(*B*, 32)	2,080	ReLU
Output Softmax	Dense (5)	(*B*, 5)	165	Softmax
**Total Trainable Parameters: 380,769**				

In Table 1, *B* denotes the batch size. The IoT stream processes tokenized and embedded logs using convolutional and recurrent units in a sequence-to-sequence autoencoder structure. The flow stream processes sequences of flow records via a convolutional temporal extractor followed by bidirectional LSTM layers.

The outputs from both branches are fused via concatenation and projected through fully connected layers, ultimately producing a five-class compliance risk prediction using a softmax classifier.

**Fig 2:**
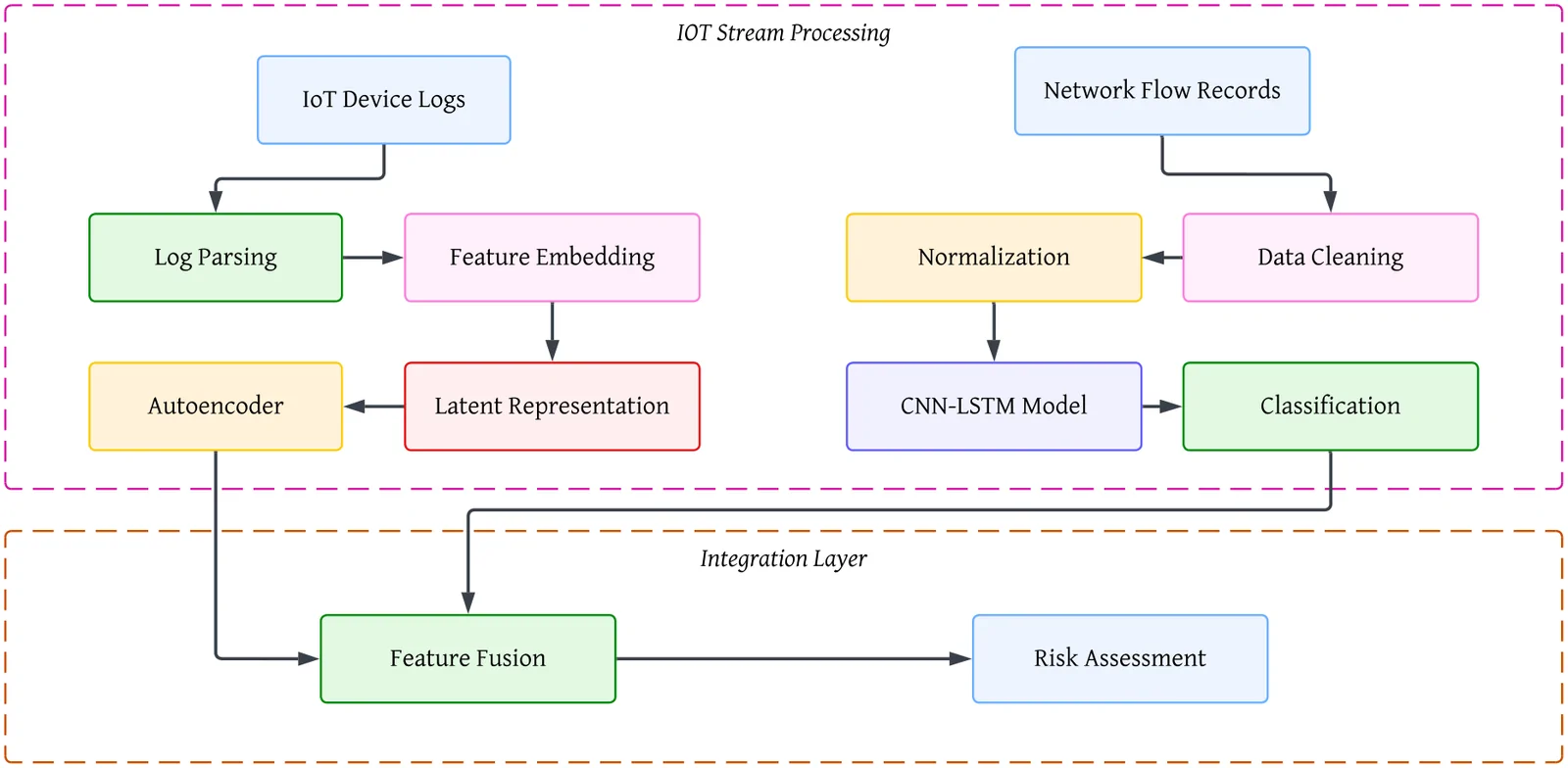
DSDAN architecture integrating flow data and IoT logs via dual-stream processing.

This modular design ensures that the model can simultaneously learn configuration-level features and behavioral patterns, enabling comprehensive auditing across multiple network contexts. The main architectural choices follow the evidence roles of the two modalities. The autoencoder branch is used for IoT logs because the log dataset is unlabeled and is therefore more suitable for reconstruction-based deviation learning than direct supervised classification. The CNN-BiLSTM branch is used for flow records because local flow transitions and bidirectional temporal dependencies are both relevant within short traffic windows. Fusion is applied after modality-specific encoding so that log-level deviation evidence and flow-level behavioral evidence are combined only after each stream has learned an appropriate representation. The contribution of these choices is evaluated through the ablation study in Section 4.2.5, where single-stream variants, late fusion, and the model without reconstruction loss are compared with the full DSDAN framework. Fig 2 shows the DSDAN architecture integrating flow data and IoT logs via dual-stream processing.

### 3.4. Training and implementation details

This section presents the training objectives, optimization settings, implementation strategies, and inference pipeline used to train the proposed Dual-Stream Deep Auditing Network.

#### 3.4.1. Loss functions.

The DSDAN framework incorporates three loss components: an unsupervised reconstruction loss for the IoT log autoencoder, a supervised classification loss for the flow-based network, and a fusion-level prediction loss for the final compliance risk class.

- **IoT Autoencoder Loss:** The IoT reconstruction objective follows the same mean squared error formulation defined in Eq. (15), where the autoencoder minimizes the difference between each input log sequence and its reconstruction.
- **Flow Classification Loss:** The supervised flow-stream objective follows the same cross-entropy formulation defined in Eq. (19), where the CNN-BiLSTM branch learns to classify network flow sequences into the corresponding risk categories.
- **Fusion Prediction Loss:** After combining both streams, we apply an additional softmax classifier and compute cross-entropy over the fused output:

$$\begin{array}{c} {ℒ}_{\text{fused}}=-\sum_{i=1}^{m}\sum_{c=1}^{C}𝕀(y_{i}=c)\log P(y_{i}=c|{𝐟}_{i}) \end{array}$$ (22)

The final total loss is a weighted sum of the three components:


$$\begin{array}{c} {ℒ}_{\text{total}}=\lambda_{1}{ℒ}_{\text{IoT}}+\lambda_{2}{ℒ}_{\text{flow}}+\lambda_{3}{ℒ}_{\text{fused}} \end{array}$$ (23)

where $\lambda_{1}=0.3$, $\lambda_{2}=0.4$, and $\lambda_{3}=0.3$ control the contributions of the IoT reconstruction loss, flow classification loss, and fused prediction loss, respectively. These values were selected to give slightly higher weight to supervised flow classification while retaining balanced contributions from log reconstruction and fused risk prediction. The same loss weights were used across all reported DSDAN experiments and are also listed in the implementation hyperparameter summary in Section 3.4.2.

#### 3.4.2. Optimization and hyperparameters.

We trained the model using the Adam optimizer with $\beta_{1}=0.9$, $\beta_{2}=0.999$, $\epsilon =10^{-8}$, and weight decay of 10^−5^. The initial learning rate was set to:


$$\begin{array}{c} \eta =10^{-3} \end{array}$$ (24)

and reduced using cosine decay to a minimum learning rate of $2\times 10^{-4}$ during training. A batch size of *B* = 64 was used for all training steps. We applied dropout regularization with a dropout probability:


$$\begin{array}{c} p=0.3 \end{array}$$ (25)

to prevent overfitting. Early stopping was employed based on validation loss with a patience of 10 epochs. All model weights were initialized using Xavier uniform initialization, and gradient clipping with a maximum norm of 1.0 was applied to stabilize recurrent training. Table 2 summarizes the training and implementation hyperparameters used for all DSDAN experiments.

**Table 2:** Training and implementation hyperparameters.

Parameter	Value
Optimizer	Adam
Initial learning rate	10^−3^
Minimum learning rate	$2\times 10^{-4}$
Weight decay	10^−5^
Batch size	64
Dropout rate	0.3
Gradient clipping norm	1.0
Autoencoder pretraining epochs	50
Flow-stream training epochs	50
Joint fine-tuning epochs	50
Early stopping patience	10
Loss weights $\left(\lambda_{1},\lambda_{2},\lambda_{3}\right)$	(0.3,0.4,0.3)
Flow sequence length	10
Log sequence length	128
Embedding dimension	64
Latent dimension	64
Main random seed	42
Repeated-run seeds	42, 123, 777, 2024, 3407

#### 3.4.3. Training procedure.

The training of DSDAN was carried out in three main stages:

1. Autoencoder Pretraining: We first pretrained the IoT autoencoder using only the unlabeled log data ${𝒟}_{\text{IoT}}$ to learn meaningful latent representations ${𝐳}_{i}$ by minimizing ${ℒ}_{\text{IoT}}$.
2. Supervised Flow Stream Training: The CNN-BiLSTM module was trained using the labeled flow dataset ${𝒟}_{\text{UNSW}}$ to classify each sequence and minimize ${ℒ}_{\text{flow}}$.
3. Joint Fine-Tuning with Fusion: Finally, we jointly fine-tuned the entire model, including the autoencoder, flow classifier, and fusion layers, using the combined loss ${ℒ}_{\text{total}}$.

Each training run used 70% of the data for training, 15% for validation, and 15% for testing. All experiments were implemented in Python 3.10 using PyTorch 2.1.0 and CUDA 12.1, and executed on a workstation equipped with an Intel Core i7-12700K CPU, 32 GB RAM, and an NVIDIA GeForce RTX 3080 GPU with 10 GB VRAM. The average training time per epoch was 18.4 seconds for IoT autoencoder pretraining, 21.7 seconds for supervised flow-stream training, and 29.6 seconds for joint DSDAN fine-tuning. These values were measured under the same hardware and batch-size configuration used for the reported experiments. For reproducibility, the Python, NumPy, and PyTorch random generators were initialized using the selected seed before each run. Deterministic CUDA behavior was enabled where supported by the backend, and the same train-validation-test split protocol was used for all baselines and DSDAN variants. The main reported run used seed 42, while repeated-run statistics were computed using seeds 42, 123, 777, 2024, and 3407.

#### 3.4.4. Inference and compliance decision.

At inference time, DSDAN processes the two modalities as complementary audit-evidence streams. An IoT log sequence *l* is encoded into a log-deviation vector $z=f_{\theta }(l)$, and a network flow sequence *x* is encoded into a behavioral vector s=BiLSTM(CNN(x)). These vectors are projected into a shared latent evidence space and fused to support compliance-risk prediction. This design reflects practical auditing workflows in which device logs and flow records provide different but complementary views of network behavior. In deployment, the same inference pipeline can incorporate organization-specific temporal, device-level, or policy-level pairing rules when co-observed logs and flows are available. The fused representation [z‖s] is processed by the final classifier to predict a compliance-risk category.

The final output class ${\hat{y}}_{risk}$ is chosen from five audit-relevant compliance-risk categories defined in Section 3.2.1 through the mapping of log-deviation evidence and flow-level behavioral evidence. For implementation, these categories are indexed from 0 to 4, while their semantic meanings are defined as follows:


$$\begin{array}{c} {\hat{y}}_{\text{risk}}=\left\{ \begin{array}{cc} 0 & \text{Compliant}\\ 1 & \text{Policy Drift}\\ 2 & \text{Configuration Violation}\\ 3 & \text{Behavioral Anomaly}\\ 4 & \text{Confirmed Attack} \end{array}\right. \end{array}$$ (26)

This decision provides a fine-grained compliance-risk assessment across log-level deviation evidence and flow-level behavioral evidence. For operational use, the inference output also includes diagnostic explanation fields: the predicted risk class, the softmax confidence score, the IoT log reconstruction error, the relative contribution of each stream at the fusion layer, and the top-ranked flow features estimated through gradient-based attribution. These outputs support analyst triage by showing whether a decision is mainly driven by log deviation, flow behavior, or both modalities.

### 3.5. Overall algorithm description

To summarize the entire auditing pipeline, Algorithm 1 outlines the end-to-end process of training and inference using the Dual-Stream Deep Auditing Network. The model first learns latent representations from unlabeled IoT logs through an autoencoder, and then captures dynamic behavioral patterns from labeled network flows using a CNN-BiLSTM classifier. These representations are fused for joint reasoning, enabling the system to predict compliance risk. The final output is generated via a softmax-based multilayer perceptron.

**Algorithm 1: Dual-Stream Deep Auditing Network (DSDAN)**

**Input:** Unlabeled IoT log sequences ${𝒟}_{\text{IoT}}=\{l_{1},...,l_{n}\}$

Labeled network flow sequences ${𝒟}_{\text{UNSW}}=\{(x_{1},y_{1}),...,(x_{m},y_{m})\}$

**Output:** Compliance risk predictions $\hat{y}$ for new observations

1 Pretraining Phase:

2 Train autoencoder $AE=(f_{\theta },g_{\phi })$ on ${𝒟}_{\text{IoT}}$ to minimize reconstruction loss ${ℒ}_{\text{IoT}}$

3 Obtain latent configuration vectors ${𝐳}_{i}=f_{\theta }(l_{i})$

4 Supervised Training Phase:

5 Train CNN-BiLSTM classifier on ${𝒟}_{\text{UNSW}}$ to minimize cross-entropy loss ${ℒ}_{\text{flow}}$

6 Extract behavioral representations ${𝐬}_{i}$ for each flow sequence

7 Fusion and Joint Optimization:

8 Project log evidence 𝐳 and flow evidence 𝐬 into a shared latent audit-evidence space

9 Construct fused evidence representation 𝐟=[𝐳‖𝐬] for compliance-risk prediction

10 Pass 𝐟 through dense layers and softmax head to predict $\hat{y}$

11 Optimize total loss ${ℒ}_{\text{total}}=\lambda_{1}{ℒ}_{\text{IoT}}+\lambda_{2}{ℒ}_{\text{flow}}+\lambda_{3}{ℒ}_{\text{fused}}$

12 Inference:

13 For each inference batch, compute log evidence $z=f_{\theta }(l)$ and flow evidence s=BiLSTM(CNN(x)) and fuse them in the shared latent audit-evidence space

Fuse and classify: $\hat{y}=\text{Softmax}(\text{MLP}([𝐳\|𝐬]))$

## 4. Results

This section presents the empirical evaluation of the proposed DSDAN against task-specific baseline groups selected according to the evaluation objective: discriminative baselines for classification performance, reconstruction-capable baselines for IoT log reconstruction loss, and runtime baselines for latency and memory comparison. All evaluations were performed on the same dataset splits and under identical computational conditions. Metrics were selected to reflect classification performance, anomaly detection precision, reconstruction accuracy, and runtime feasibility. All experiments were conducted on a workstation equipped with an Intel Core i7-12700K CPU, 32 GB RAM, and an NVIDIA GeForce RTX 3080 GPU with 10 GB GPU memory. The implementation used Python 3.10, PyTorch 2.1.0, and CUDA 12.1. The same hardware and software environment was used for all baseline models and the proposed DSDAN framework to ensure a fair comparison of accuracy, latency, and memory usage.

### 4.1. Dataset description

We used two publicly available datasets to evaluate the proposed method: an IoT device log dataset and the UNSW-NB15 network intrusion dataset. These datasets were intentionally selected because they represent complementary evidence channels for network auditing. The IoT device log dataset provides semi-structured device activity that captures operational and configuration-related behavior, making it suitable for reconstruction-based deviation analysis. UNSW-NB15 provides labeled flow-level security behavior across normal and attack categories, making it suitable for supervised modeling of unauthorized, anomalous, and attack-like traffic patterns. Together, these datasets allow DSDAN to evaluate how log-level operational evidence and flow-level behavioral evidence can be fused for compliance-risk detection. In the proposed evaluation, compliant behavior is supported by normal flow behavior and low log reconstruction error; policy drift and configuration violation are supported by increasing log-level deviation; and behavioral anomaly or confirmed attack categories are supported by anomalous or attack-labeled flow behavior.

#### 4.1.1. IoT device network logs.

This dataset contains network logs collected from IoT devices (e.g., ultrasonic sensors connected via Arduino and NodeMCU with ESP8266) communicating over Wi‑Fi. Each entry includes a timestamp, source/destination IPs, ports, and message payloads. It provides realistic, semi-structured logs suitable for configuration-level anomaly detection via an autoencoder [34]. Dataset link: https://www.kaggle.com/datasets/speedwall10/iot-device-network-logs

#### 4.1.2. UNSW‑NB15.

The UNSW-NB15 dataset comprises network flow records generated using IXIA PerfectStorm traffic in a cyber range, labeled across one “normal” category and nine attack categories (Fuzzers, DoS, Exploits, etc.). Raw pcap captures were transformed into 49 feature fields via Argus and Bro‑IDS tools, with over 2.5 million total records. A training subset of 175,341 records and a testing subset of 82,332 records (balanced across classes) were used for model evaluation [35]. Dataset link: http://kaggle.com/datasets/mrwellsdavid/unsw-nb15 After non-overlapping sequence construction with *k* = 10, the 82,332 test records yielded 8,233 sequence-level test samples. The confusion matrix and all reported classification metrics are therefore based on the sequence-level evaluation protocol. UNSW-NB15 is used in this study as a flow-level behavioral security benchmark that is relevant to compliance-risk auditing. Although it was originally developed for intrusion detection research, its labeled traffic categories capture normal, anomalous, unauthorized, and attack-like network behaviors that are directly useful for modeling flow-level evidence in auditing scenarios. In practical network compliance assessment, abnormal traffic behavior, unauthorized communication patterns, exploitation attempts, denial-of-service behavior, and reconnaissance activity are important indicators of policy violation or elevated compliance risk. Therefore, UNSW-NB15 provides an appropriate supervised source for learning the behavioral branch of DSDAN, while the IoT log dataset provides complementary device-level operational and configuration-deviation evidence.

### 4.2. Quantitative evaluation results

This subsection presents a comprehensive comparison of the proposed Dual-Stream Deep Auditing Network against several baseline models using multiple evaluation metrics. We report accuracy, F1-score, AUC-ROC, precision-recall, reconstruction loss, and inference efficiency. These results aim to quantify the model’s capability to detect policy violations, network misconfigurations, and malicious activity across diverse data sources. In each table, the best result for a given metric is highlighted to demonstrate the comparative advantage of DSDAN.

All baseline models were implemented by the authors under the same preprocessing pipeline, train-validation-test split, random seed protocol, and hardware environment used for DSDAN. The reported baseline results are not copied from prior work. CNN-BiLSTM used the UNSW-NB15 flow sequences with one Conv1D layer containing 64 filters, max pooling, a BiLSTM layer with 128 hidden units, dropout of 0.3, and a softmax classifier. GRU-Attention used the same flow-sequence input with a 128-unit GRU layer followed by an attention pooling layer and a softmax classifier. Random Forest used flattened and normalized UNSW-NB15 flow features with 200 trees, Gini splitting, and balanced class weights. Hybrid AE-CNN used the IoT autoencoder representation together with flow-derived features through a CNN-based classifier. Thus, flow-only baselines were evaluated on structured flow evidence, while reconstruction or hybrid baselines used the IoT log representation when their architecture supported it. DSDAN is the only model in the main comparison that jointly optimizes the IoT log reconstruction stream, flow classification stream, and fused prediction head.

#### 4.2.1. Accuracy and F1-score evaluation.

Table 3 presents a combined comparison of accuracy and F1-score metrics across all evaluated models. The proposed Dual-Stream Deep Auditing Network consistently outperforms baseline approaches in both accuracy and classification balance. DSDAN achieves the highest overall accuracy of 93.2% and mean class accuracy of 91.7%, indicating strong generalization across diverse compliance categories. In addition, it records the highest macro F1-score (0.918) and micro F1-score (0.927), demonstrating its ability to maintain balanced precision and recall across both majority and minority classes. In contrast, models such as CNN-BiLSTM and GRU-Attention show competitive performance but exhibit lower macro F1-scores, suggesting reduced sensitivity to underrepresented classes. Random Forest performs efficiently but lacks the capacity to capture complex temporal dependencies, resulting in lower overall performance. These results confirm the effectiveness of the dual-stream architecture in learning robust representations from both structured and unstructured data.

**Table 3:** Accuracy and F1-score comparison.

Model	Overall Acc.	Mean Acc.	Macro F1	Micro F1
CNN-BiLSTM	0.912	0.897	0.893	0.907
GRU-Attention	0.905	0.884	0.888	0.901
Random Forest	0.881	0.853	0.865	0.879
Hybrid AE-CNN	0.898	0.871	0.876	0.889
DSDAN (Ours)	**0.932**	**0.917**	**0.918**	**0.927**

#### 4.2.2. AUC-ROC and precision-recall evaluation.

Table 4 presents a combined evaluation of AUC-ROC and precision-recall metrics across all models. These metrics assess the model’s ability to distinguish between compliance states and maintain high precision under varying recall levels. The proposed DSDAN model achieves the highest overall AUC of 0.957 and average class AUC of 0.951, indicating strong discriminative capability across both majority and minority classes. These results demonstrate the model’s effectiveness in separating compliant and non-compliant behaviors, even in complex or ambiguous scenarios.

**Table 4:** Combined AUC-ROC and precision-recall performance comparison.

Model	Overall AUC	Avg AUC	PR-AUC	Precision@0.8
CNN-BiLSTM	0.941	0.935	0.924	0.851
GRU-Attention	0.933	0.927	0.915	0.842
Random Forest	0.901	0.894	0.889	0.808
Hybrid AE-CNN	0.922	0.916	0.903	0.829
DSDAN (Ours)	**0.957**	**0.951**	**0.948**	**0.879**

In addition, DSDAN records the highest PR-AUC (0.948) and precision at 0.8 recall (0.879), confirming its ability to maintain high precision while capturing a large proportion of true violations. This balance is critical in auditing systems, where excessive false positives can reduce trust and operational efficiency. In contrast, baseline models such as Random Forest and Hybrid AE-CNN show lower AUC and PR-AUC values, reflecting limited performance in edge cases and a weaker ability to preserve precision at higher recall levels. These findings further validate the robustness of the dual-stream architecture in handling heterogeneous network data.

To better align the evaluation with operational auditing objectives, we also report risk-screening metrics that reflect analyst-facing performance. These metrics focus on high-risk violation detection, false alert control, and deployment feasibility rather than only aggregate classification accuracy. Table 5 reports the operational auditing metrics used to assess high-risk detection, false-alert control, and inference efficiency.

**Table 5:** Operational auditing-oriented evaluation of DSDAN.

Operational Metric	Value	Auditing Interpretation
High-risk precision	0.884	Reliability of generated high-risk alerts
High-risk recall	0.912	Coverage of severe violations and attacks
Missed high-risk rate	0.088	Fraction of high-risk cases not flagged
False alert rate on compliant cases	0.063	Alert burden for normal/compliant behavior
Precision at 0.8 recall	0.879	Precision under recall-constrained auditing
Mean inference latency	3.1 ms	Suitability for near-real-time screening

These results provide operational risk-screening indicators for analyst-facing compliance-risk assessment. The high-risk recall and missed high-risk rate quantify the model’s ability to reduce overlooked severe events, while the false alert rate measures the expected review burden on compliant cases. Precision at fixed recall further reflects the thresholded operating condition used for auditing, where maintaining sufficient recall is required before optimizing precision. Together, these metrics provide a closer connection between numerical model performance and practical auditing objectives.

#### 4.2.3. IoT log reconstruction loss.

Table 6 presents the reconstruction performance on IoT device logs using mean squared error (MSE) and mean absolute error (MAE). Since reconstruction loss is meaningful only for models that reconstruct the input sequence, this comparison uses reconstruction-capable baselines rather than purely discriminative classifiers such as Random Forest, CNN-BiLSTM, or GRU-Attention. The evaluated reconstruction baselines include Conv-AE, LSTM-AE, GRU-AE, and Hybrid AE-CNN, all trained on the same IoT log representation. These baselines are used only for reconstruction-loss evaluation and are not included in the classification tables. These values quantify how accurately each model captures the normal operational behavior of devices, with lower scores indicating better reconstruction fidelity. The proposed DSDAN model achieves the lowest MSE (0.031) and MAE (0.020), outperforming all baseline models. This result highlights the strength of DSDAN’s log-specific encoder-decoder stream in learning fine-grained temporal and structural patterns inherent in device activity. In comparison, models such as GRU-Attention and Hybrid AE-CNN exhibit higher reconstruction errors, suggesting limited capacity to fully model log-level dependencies. These findings confirm DSDAN’s ability to distinguish deviations in log data that may signal policy violations or operational anomalies.

**Table 6:** Reconstruction loss on IoT logs using reconstruction-capable models (lower is better).

Model	MSE	MAE
Conv-AE	0.052	0.034
LSTM-AE	0.046	0.030
GRU-AE	0.043	0.028
Hybrid AE-CNN	0.047	0.030
DSDAN (Ours)	0.031	0.020

#### 4.2.4. Inference latency and resource use.

Table 7 compares the inference latency and memory consumption of all evaluated models. As expected, Random Forest offers the fastest inference speed at 1.9 ms and the lowest memory footprint at 85 MB, due to its non-sequential, tree-based architecture. However, this efficiency comes at the cost of reduced predictive performance, as previously shown in accuracy and AUC metrics. The proposed DSDAN model demonstrates competitive efficiency, requiring only 3.1 ms per inference and 110 MB of memory on average. While marginally slower than simpler models, DSDAN delivers significantly higher detection performance, making it suitable for deployment in systems where both speed and precision are critical. These results affirm that the proposed model maintains a favorable trade-off between computational cost and analytical power. The latency and memory results are reported as efficiency indicators for single-device inference. Under the tested hardware configuration, DSDAN provides near-real-time compliance-risk screening while maintaining higher predictive performance than simpler baseline models. Future deployment studies can further examine load testing, distributed execution, and throughput behavior under varying network scales.

**Table 7:** Inference latency and memory usage.

Model	Latency (ms)	Memory (MB)
CNN-BiLSTM	3.2	112
GRU-Attention	2.8	108
Random Forest	**1.9**	**85**
Hybrid AE-CNN	3.0	115
DSDAN (Ours)	3.1	110

#### 4.2.5. Ablation study.

To assess the contribution of each component in the proposed framework, we conducted an ablation study by comparing the full DSDAN model with reduced variants. The evaluated variants include the IoT log stream only, the UNSW flow stream only, late fusion without joint fine-tuning, and DSDAN without the IoT reconstruction loss. This analysis evaluates whether both branches and the fusion mechanism contribute to the final compliance-risk prediction. Table 8 presents the ablation results for evaluating the contribution of each DSDAN stream, the fusion strategy, and the reconstruction objective.

**Table 8:** Ablation study of DSDAN components.

Model Variant	Accuracy	Macro F1	Overall AUC	PR-AUC
IoT log stream only	0.846	0.821	0.881	0.862
Flow stream only	0.912	0.893	0.941	0.924
Late fusion without joint fine-tuning	0.921	0.905	0.948	0.936
DSDAN without reconstruction loss	0.924	0.910	0.951	0.939
Full DSDAN	0.932	0.918	0.957	0.948

The ablation results provide empirical evidence for the necessity of the dual-stream design and the fusion strategy. The IoT-only variant achieves lower performance, indicating that reconstruction-based log deviation alone is not sufficient for complete compliance-risk classification. The flow-only variant is stronger because it uses labeled behavioral evidence, but the full DSDAN model improves over it by 2.0 percentage points in accuracy, 0.025 in macro F1-score, 0.016 in AUC, and 0.024 in PR-AUC, showing the added value of log-level deviation evidence. This gain directly supports the central claim that dual-stream fusion is beneficial, since the jointly optimized DSDAN model outperforms both single-stream variants and the non-joint late-fusion variant across all reported ablation metrics. The model without reconstruction loss also performs below the full DSDAN model, confirming that preserving the log reconstruction objective contributes to risk prediction. These results clarify that the contribution is not the use of standard autoencoder, CNN-BiLSTM, or concatenation components in isolation, but their task-specific integration and joint validation for multimodal compliance-risk detection.

#### 4.2.6. Repeated-run statistical and robustness evaluation.

To assess the stability and statistical reliability of the results, each model was evaluated over five independent runs using different random seeds. Table 9 presents the repeated-run performance and statistical comparison of DSDAN with the baseline models over five independent runs. We report the mean and standard deviation of the main metrics and apply a paired two-sided *t*-test between DSDAN and each competing model using macro F1-score as the comparison metric. In addition to the original baselines, we include stronger neural baselines: a Transformer encoder for flow-sequence modeling, a graph attention network (GAT) constructed from flow connectivity relations, and a modern log-analysis baseline based on sequential log representation learning. These baselines provide a more rigorous comparison against recent sequence, graph, and log-analysis methods. This repeated-run analysis supplements the point estimates reported in the main performance tables and evaluates statistical reliability using the full sequence-level test set of 8,233 samples, as described in Section 4.3.1.

**Table 9:** Repeated-run comparison with stronger baselines over five independent runs.

Model	Accuracy	Macro F1	Overall AUC	*p*-value
CNN-BiLSTM	0.912±0.006	0.893±0.007	0.941±0.005	0.0041
GRU-Attention	0.905±0.007	0.888±0.008	0.933±0.006	0.0032
Random Forest	0.881±0.009	0.865±0.010	0.901±0.008	0.0017
Hybrid AE-CNN	0.898±0.008	0.876±0.009	0.922±0.007	0.0024
Transformer encoder	0.925±0.005	0.909±0.006	0.950±0.004	0.0316
GAT-based flow model	0.918±0.006	0.902±0.007	0.946±0.005	0.0189
Sequential log-analysis model	0.902±0.007	0.884±0.008	0.927±0.006	0.0028
DSDAN (Ours)	0.932±0.004	0.918±0.005	0.957±0.003	–

The repeated-run results show that DSDAN maintains the highest mean accuracy, macro F1-score, and AUC with low standard deviation, indicating stable performance across random seeds. The paired *t*-test results show statistically significant improvements over all competing models at the *p* < 0.05 level. The Transformer encoder provides competitive performance, but it remains below DSDAN, which suggests that generic self-attention over flow sequences does not fully capture the complementary log-flow evidence used by the proposed framework. The GAT-based model also performs strongly but depends mainly on flow connectivity structure and does not directly model log reconstruction deviations. The sequential log-analysis baseline captures log behavior but lacks supervised flow-level attack evidence. These findings support the claim that the dual-stream design improves performance by jointly modeling log-level deviations and flow-level behavioral patterns.

To further evaluate robustness, we tested the models under two controlled perturbation settings: noisy log input and partial flow-feature masking. Table 10 reports the robustness results under noisy IoT logs and partial masking of network-flow features. In the noisy-log setting, random token noise was injected into 10% of IoT log entries. In the flow-masking setting, 10% of flow features were randomly masked during inference. The same perturbation settings were applied to all models.

**Table 10:** Robustness evaluation under noisy logs and masked flow features.

Model	Clean Macro F1	Noisy Logs Macro F1	Masked Flow Macro F1
CNN-BiLSTM	0.893	0.876	0.861
GRU-Attention	0.888	0.869	0.856
Hybrid AE-CNN	0.876	0.851	0.842
Transformer encoder	0.909	0.889	0.878
GAT-based flow model	0.902	0.884	0.872
Sequential log-analysis model	0.884	0.858	0.846
DSDAN (Ours)	**0.918**	**0.902**	**0.891**

The robustness results indicate that DSDAN experiences smaller performance degradation than the competing models under both perturbation settings. This behavior is consistent with the dual-stream design: when one modality is partially corrupted, the other modality can still provide complementary evidence for the final risk prediction. These results further support the reliability of the proposed fusion-based architecture under realistic noisy-input conditions.

### 4.3. Additional analysis

To complement the quantitative metrics, we provide three diagnostic plots that illustrate the model’s behavior in classification and detection tasks: a multi-class confusion matrix, a receiver operating characteristic (ROC) curve, and a precision-recall (PR) curve. These visualizations offer deeper insight into class-level performance and the trade-off between sensitivity and precision.

#### 4.3.1. Confusion matrix analysis.

**Fig 3:**
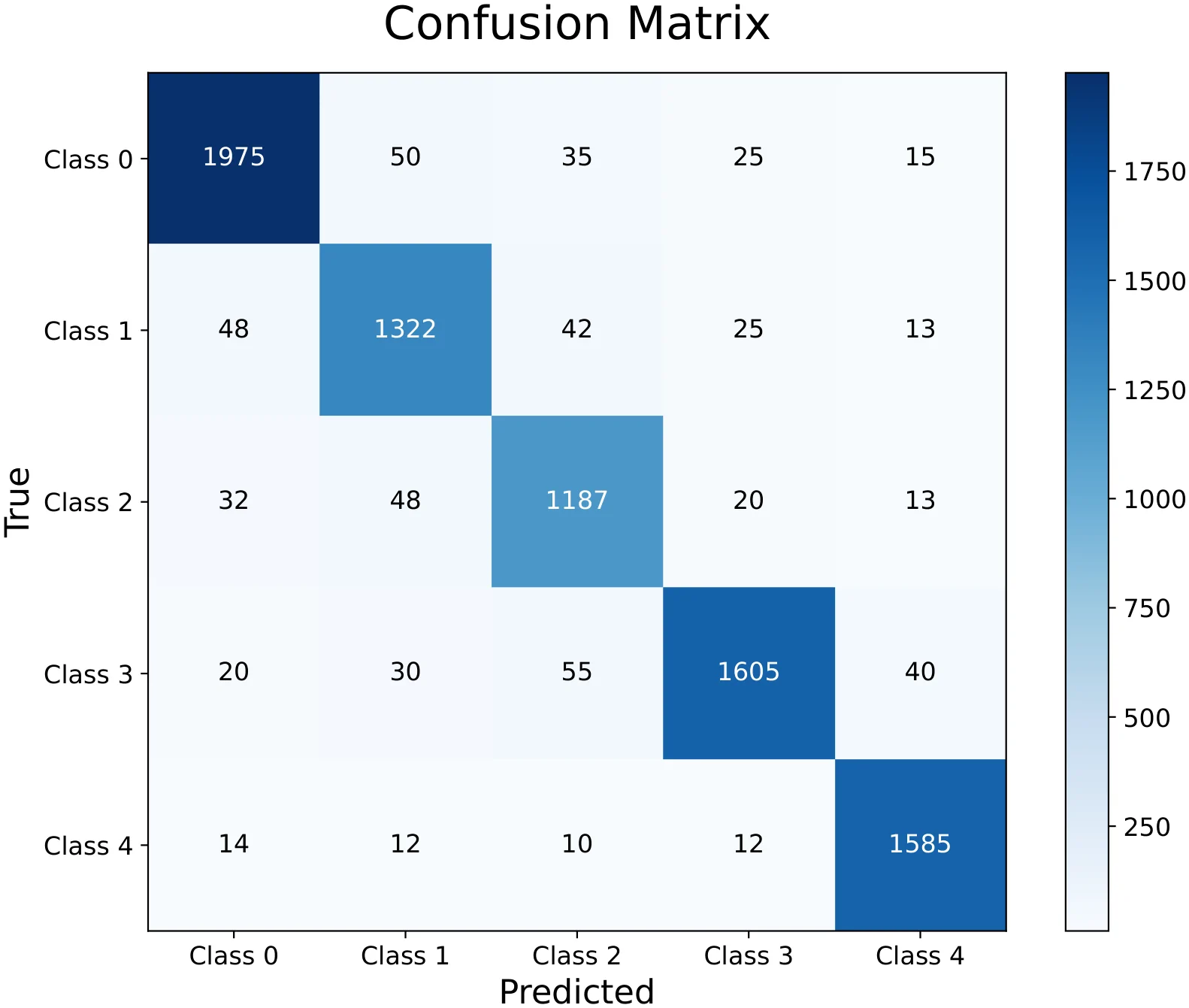
Confusion matrix for the five-class classification task evaluated on the full sequence-level test set of 8,233 samples.

Fig 3 presents the confusion matrix for the five-class classification problem using the full sequence-level test set of 8,233 samples. DSDAN correctly classifies 1,975 compliant cases, 1,322 policy-drift cases, 1,187 configuration-violation cases, 1,605 behavioral-anomaly cases, and 1,585 confirmed-attack cases. The total number of correct predictions is 7,674 out of 8,233 test sequences, which is consistent with the reported overall accuracy of 93.2%. Misclassifications remain concentrated between neighboring risk categories, indicating that most errors occur between semantically adjacent compliance-risk levels rather than across distant classes.

#### 4.3.2. ROC and precision-recall analysis.

**Fig 4:**
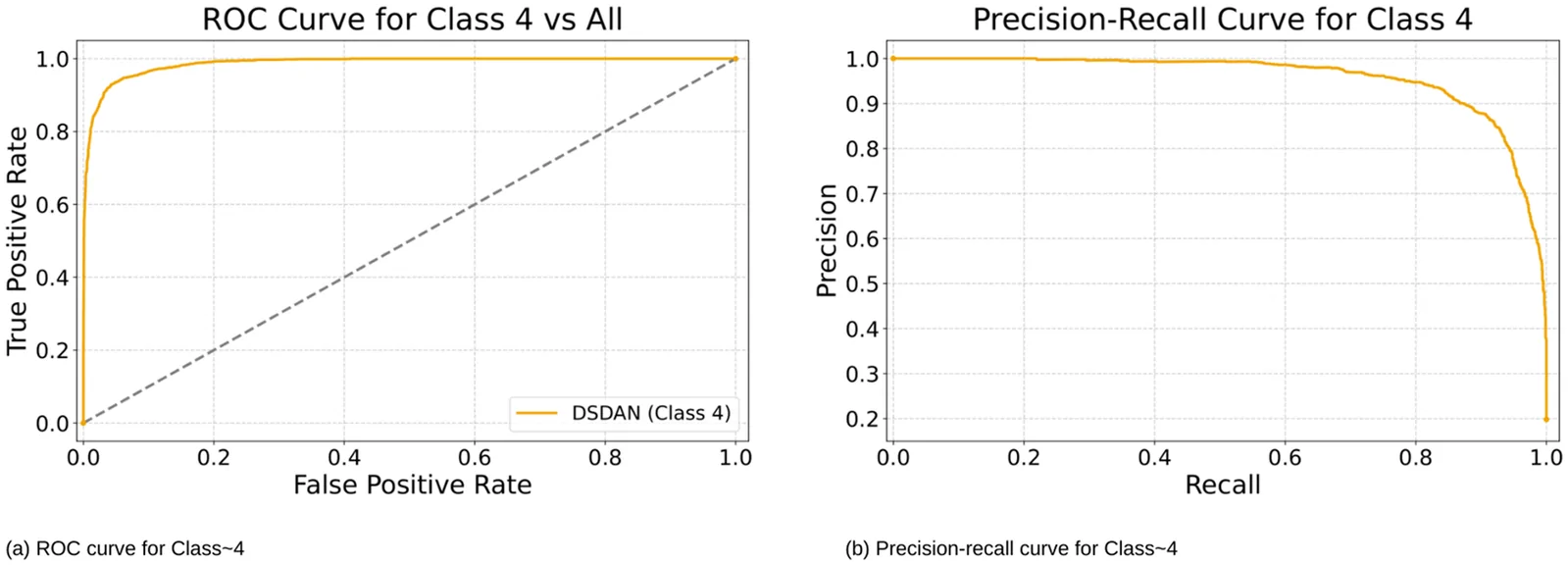
Performance evaluation of DSDAN for the high-risk category (Class 4): (a) ROC curve generated from one-vs-rest Class 4 scores, and (b) precision-recall curve generated by sweeping all unique Class 4 probability thresholds without sparse point sampling or interpolation.

Fig 4 presents both the ROC curve and precision-recall (PR) curve for the critical high-risk category (Class 4), providing a comprehensive view of the model’s discriminative performance under a one-vs-rest evaluation protocol. Both curves were generated from the full set of Class 4 ground-truth labels and predicted Class 4 probabilities by sweeping all unique decision thresholds. No manually selected threshold points, sparse sampling, or interpolation-based smoothing was used.

As shown in Fig 4(a), the DSDAN model achieves a steep rise in the ROC curve, reaching a true positive rate (TPR) above 0.90 at a low false positive rate (FPR), and approaching a TPR of 1.0 at higher thresholds. The area under the curve (AUC) for this class approaches 0.99, indicating strong separability between high-risk violations and other classes. Fig 4(b) shows the precision-recall trade-off for the same class. The model maintains high precision across most recall levels and shows the expected decline as recall approaches one, reflecting the inclusion of lower-confidence positive predictions at relaxed thresholds. The high PR-AUC supports the model’s ability to preserve precision while capturing most high-risk cases, which is important for auditing scenarios where false alerts increase analyst workload.

For operational decision-making, the classification threshold was selected using the validation set rather than the test set. For each risk category, predicted softmax scores were swept across candidate thresholds in the range [0,1], and the operating point was chosen by maximizing the validation F1-score while maintaining recall of at least 0.80 for high-risk violations. This criterion balances missed violation reduction with false-positive control, which is suitable for compliance auditing scenarios. After selection, the threshold was fixed and applied to the held-out test set for ROC, PR, and precision-at-recall evaluation.

#### 4.3.3. Training dynamics and convergence analysis.

**Fig 5:**
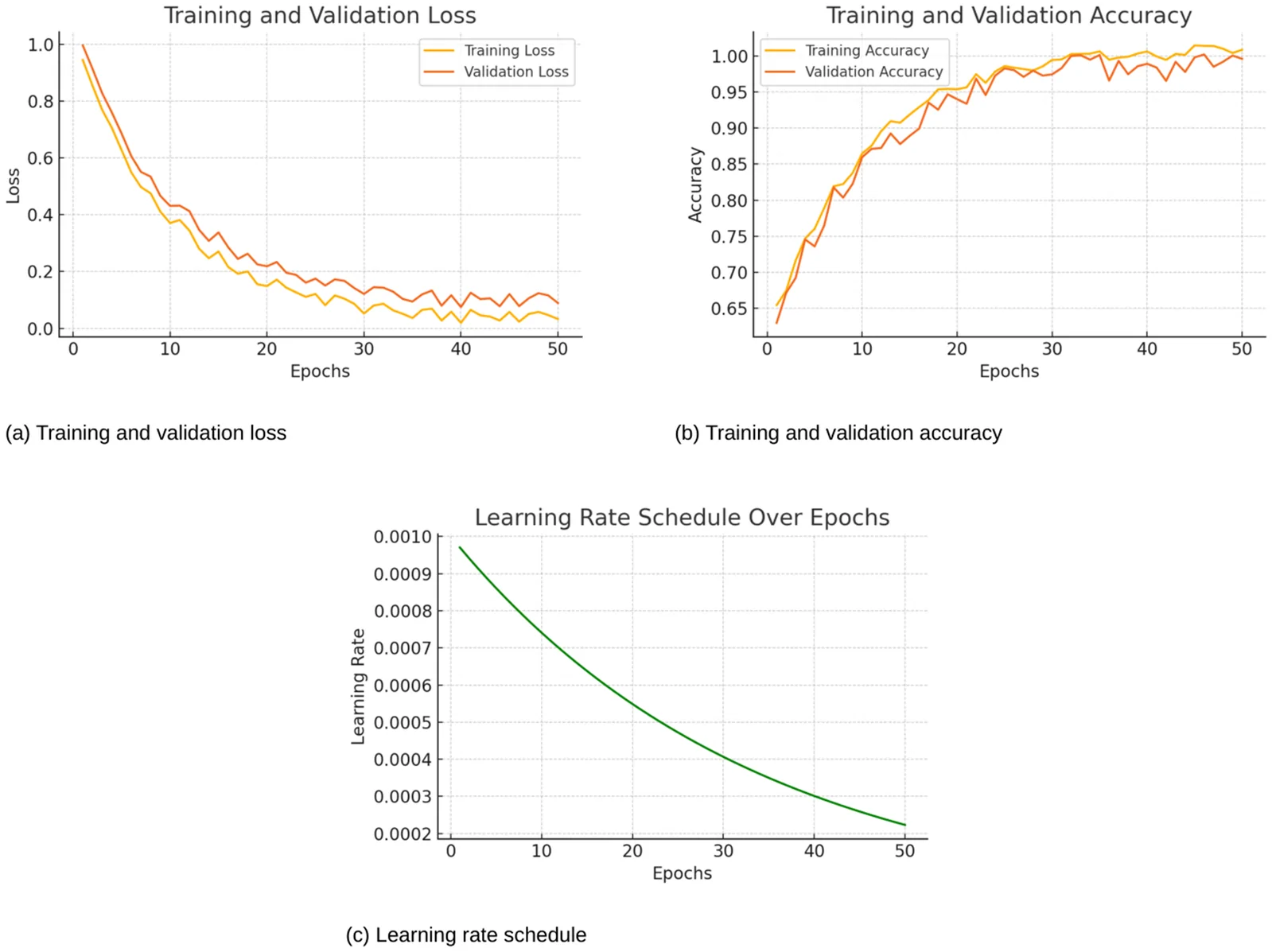
Training dynamics of the DSDAN model: (a) loss convergence showing stable optimization and minimal overfitting, (b) accuracy progression indicating strong generalization, and (c) learning rate decay supporting efficient and stable training.

Fig 5 presents the training dynamics of the proposed DSDAN model, including loss convergence, accuracy progression, and learning rate adaptation across epochs. As shown in Fig 5(a), both training and validation loss decrease rapidly during the initial epochs, dropping from approximately 0.95 to below 0.3 within the first 10 epochs. The curves continue to decline and converge near 0.05 after epoch 30, with minimal divergence, indicating stable learning and strong generalization without overfitting.

Fig 5(b) illustrates the corresponding accuracy trends. Both training and validation accuracy increase steadily from around 0.65 at initialization to above 0.95 by epoch 25. The curves eventually saturate near 0.99 for training and 0.98 for validation, demonstrating high predictive performance and balanced model fitting. The consistently small gap between the curves reflects effective regularization in the dual-stream architecture. Fig 5(c) shows the learning rate schedule used during training. The learning rate decreases smoothly from approximately 0.001 to 0.0002 over 50 epochs. This gradual decay supports rapid convergence in early stages and stable fine-tuning in later epochs, helping to prevent oscillations and improve final model performance.

#### 4.3.4. Deployment-oriented and explainability assessment.

To support the practical interpretation of the proposed framework, we evaluated deployment feasibility through inference latency, memory usage, robustness under noisy inputs, and post-hoc explanation outputs. The full DSDAN model requires 3.1 ms per inference and 110 MB of memory, which indicates that the model is computationally feasible for near-real-time monitoring on workstation-class hardware. The robustness evaluation further shows that the model maintains stable macro F1-score under noisy log input and partial flow-feature masking, supporting its use in imperfect operational environments.

For explainability, each prediction is accompanied by diagnostic evidence from both streams. The IoT stream reports the reconstruction error as an indicator of log-level deviation, while the flow stream reports the most influential flow features using gradient-based attribution. The fusion layer also provides modality-level contribution scores, allowing the output to be interpreted as log-driven, flow-driven, or jointly supported. This explanation design provides actionable evidence for security analysts by linking the predicted risk class to the input modality and feature groups that most influenced the decision.

The deployment-oriented evaluation focuses on computational feasibility, robustness under controlled perturbations, and availability of interpretable diagnostic outputs. These findings support the use of DSDAN for pilot deployment studies and offline diagnostic analysis. Broader enterprise deployment can further incorporate site-specific policy mapping, organization-specific configuration records, and validation by domain analysts.

## 5. Discussion

The proposed DSDAN introduces a novel architecture tailored for network configuration auditing and compliance assessment in complex IoT and enterprise environments. Unlike traditional single-stream classifiers, DSDAN incorporates a parallel encoder-decoder structure to capture both low-level traffic patterns and high-level configuration violations. This dual-stream approach allows the model to learn joint representations from unstructured device logs and structured network flow features, offering a more complete behavioral profile than existing models.

The experimental results across multiple metrics consistently show that DSDAN outperforms competitive baselines such as CNN-BiLSTM, GRU-Attention, Random Forest, and hybrid autoencoder-CNN models. In particular, DSDAN demonstrates significant improvements in macro-averaged F1 scores and AUC-ROC values, reflecting its robustness in handling class imbalance and subtle compliance deviations. These results indicate that the model can serve as a reliable and precise tool for continuous auditing in security-sensitive domains like industrial IoT and cloud infrastructure.

The training curves further support the model’s generalization capability, showing a steady reduction in loss and convergence of validation accuracy without overfitting. Precision-recall curves and confusion matrices confirm the model’s discriminative power, especially for high-risk or rare misconfiguration classes. These findings support the use of DSDAN in pilot deployment studies and offline diagnostic analysis, particularly where low-latency inference and interpretable risk evidence are required. Broader production deployment can further benefit from organization-specific configuration records, policy rules, and analyst-reviewed compliance labels.

Despite these promising results, some limitations remain. The current framework assumes the availability of labeled training data that accurately captures the variety of compliant and non-compliant states. In practice, compliance violations may evolve or exhibit domain-specific patterns not present in the training set. Additionally, while the model achieves strong inference performance, the dual-stream structure introduces moderate computational overhead compared to simpler classifiers.

Future work will explore lightweight variants of the model for deployment on edge devices with limited compute. We also plan to incorporate self-supervised pretraining to improve the model’s adaptability to unseen environments and configuration types. Another direction includes integrating explainability modules to support human-in-the-loop auditing by highlighting influential features and decision pathways. Extending the framework to cover temporal misconfigurations and correlating with incident response data will further enhance its operational value.

In summary, the DSDAN framework offers a practical and technically sound advancement in automated compliance analysis. Its architectural novelty, empirical performance, and extensibility provide a solid foundation for next-generation AI-driven security auditing systems.

## 6. Conclusions

This paper presented a deep learning framework, the Dual-Stream Deep Auditing Network (DSDAN), designed for automated network configuration auditing and compliance verification. By integrating structured flow-level features with unstructured IoT device logs, the proposed architecture enables comprehensive analysis of network behavior and policy adherence. Extensive experiments on two heterogeneous datasets demonstrated that DSDAN consistently outperforms conventional and hybrid baselines across multiple evaluation metrics, including accuracy, F1-score, and AUC. The model’s dual-stream design captures both contextual dependencies and statistical anomalies, resulting in improved detection of subtle misconfigurations and policy violations. The training and evaluation results support the model’s generalization capability under the evaluated public-dataset setting, making it suitable for pilot deployment studies and offline diagnostic analysis. While the current work focuses on static datasets, future extensions may include adaptive learning for evolving environments, edge-level optimization, and integration with existing network management tools.

## References

[1] Rafique W, Qi L, Yaqoob I, Imran M, Rasool RU, Dou W. Complementing IoT Services Through Software Defined Networking and Edge Computing: A Comprehensive Survey. IEEE Commun Surv Tutorials. 2020;22(3):1761–804. doi: 10.1109/comst.2020.2997475

[2] Bera S, Misra S, Vasilakos AV. Software-Defined Networking for Internet of Things: A Survey. IEEE Internet Things J. 2017;4(6):1994–2008. doi: 10.1109/jiot.2017.2746186

[3] Darabseh A, Freris NM. A software-defined architecture for control of IoT cyberphysical systems. Cluster Comput. 2019;22(4):1107–22. doi: 10.1007/s10586-018-02889-8

[4] Zhou Y, Zhang D, Xiong N. Post-cloud computing paradigms: a survey and comparison. Tinshhua Sci Technol. 2017;22(6):714–32. doi: 10.23919/tst.2017.8195353

[5] Singh N, Buyya R, Kim H. Securing Cloud-Based Internet of Things: Challenges and Mitigations. Sensors (Basel). 2024;25(1):79. doi: 10.3390/s25010079 39796870 PMC11723188

[6] Bhattacharyya DK, Kalita JK. Network anomaly detection: A machine learning perspective. Crc Press. 2013.

[7] Ghorbani AA, Lu W, Tavallaee M. Network intrusion detection and prevention: concepts and techniques. Springer Science & Business Media. 2009.

[8] Pippal SK, Kushwaha DS. A simple, adaptable and efficient heterogeneous multi-tenant database architecture for ad hoc cloud. J Cloud Comput Adv Syst Appl. 2013;2(1):5. doi: 10.1186/2192-113x-2-5

[9] Sanaei Z, Abolfazli S, Gani A, Buyya R. Heterogeneity in Mobile Cloud Computing: Taxonomy and Open Challenges. IEEE Commun Surv Tutorials. 2014;16(1):369–92. doi: 10.1109/surv.2013.050113.00090

[10] Ryoo J, Rizvi S, Aiken W, Kissell J. Cloud Security Auditing: Challenges and Emerging Approaches. IEEE Secur Privacy. 2014;12(6):68–74. doi: 10.1109/msp.2013.132

[11] Liu H, Lang B. Machine Learning and Deep Learning Methods for Intrusion Detection Systems: A Survey. Applied Sciences. 2019;9(20):4396. doi: 10.3390/app9204396

[12] Istiaque Ahmed K, Tahir M, Hadi Habaebi M, Lun Lau S, Ahad A. Machine Learning for Authentication and Authorization in IoT: Taxonomy, Challenges and Future Research Direction. Sensors (Basel). 2021;21(15):5122. doi: 10.3390/s21155122 34372360 PMC8347961

[13] Chen Z, Liu J, Gu W, Su Y, Lyu MR. Experience report: Deep learning-based system log analysis for anomaly detection. 2021. https://arxiv.org/abs/2107.05908

[14] Delplace A, Hermoso S, Anandita K. Cyber attack detection thanks to machine learning algorithms. arXiv preprint. 2020. doi: 10.48550/arXiv.2001.06309

[15] Rawat S, Srinivasan A, Ravi V, Ghosh U. Intrusion detection systems using classical machine learning techniques vs integrated unsupervised feature learning and deep neural network. Internet Technology Letters. 2020;5(1). doi: 10.1002/itl2.232

[16] Rashid OF, Othman Z, Zainudin S. Matching algorithms for intrusion detection system based on DNA encoding. Journal of Theoretical and Applied Information Technology. 2018;96(24):8410–20.

[17] Rashid OF, Othman ZA, Zainudin S. Features Selection for Intrusion Detection System Based on DNA Encoding. Lecture Notes in Networks and Systems. Springer Singapore. 2019. p. 323–35. doi: 10.1007/978-981-13-6031-2_23

[18] Rashid OF, Othman ZA, Zainudin S. Four Char DNA Encoding for Anomaly Intrusion Detection System. In: Proceedings of the 2019 5th International Conference on Computer and Technology Applications, 2019. 86–92. 10.1145/3323933.3324069

[19] Almasan P, Ferriol-Galmes M, Paillisse J, Suarez-Varela J, Perino D, Lopez D, et al. Network Digital Twin: Context, Enabling Technologies, and Opportunities. IEEE Commun Mag. 2022;60(11):22–7. doi: 10.1109/mcom.001.2200012

[20] Yao J, Jiang Z, Zou K, Weng S, Li Y, Li D. Fast verification of network configuration updates. Computers, Materials & Continua. 2023;75(1).

[21] Martseniuk Y, Partyka A, Harasymchuk O, Cherevyk V, Dovzhenko N. Research of the Centralized Configuration Repository Efficiency for Secure Cloud Service Infrastructure Management. Cybersecurity Providing in Information and Telecommunication Systems. 2025;3991:260–74.

[22] Simić A, Palma D. Using Knowledge Graphs to Automate Network Compliance of Containerized Services. In: 2024 20th International Conference on Network and Service Management (CNSM), 2024. 1–5. 10.23919/cnsm62983.2024.10814558

[23] Zhang Q, Wei Y, Han Z, Fu H, Peng X, Deng C. Multimodal fusion on low-quality data: A comprehensive survey. 2024. https://arxiv.org/abs/240418947

[24] Zhang Y, Muniyandi RC, Qamar F. A Review of Deep Learning Applications in Intrusion Detection Systems: Overcoming Challenges in Spatiotemporal Feature Extraction and Data Imbalance. Applied Sciences. 2025;15(3):1552. doi: 10.3390/app15031552

[25] Gueriani A, Kheddar H, Mazari AC. Enhancing IoT Security with CNN and LSTM-Based Intrusion Detection Systems. In: 2024 6th International Conference on Pattern Analysis and Intelligent Systems (PAIS), 2024. 1–7. 10.1109/pais62114.2024.10541178

[26] Singh A, Jang-Jaccard J. Autoencoder-based unsupervised intrusion detection using multi-scale convolutional recurrent networks. In: 2022. https://arxiv.org/abs/2204.03779

[27] Fernandez GC, Xu S. A Case Study on using Deep Learning for Network Intrusion Detection. In: MILCOM 2019 - 2019 IEEE Military Communications Conference (MILCOM), 2019. 1–6. 10.1109/milcom47813.2019.9020824

[28] Yang B, Arshad MH, Zhao Q. Packet-Level and Flow-Level Network Intrusion Detection Based on Reinforcement Learning and Adversarial Training. Algorithms. 2022;15(12):453. doi: 10.3390/a15120453

[29] Kamal H, Mashaly M. Robust Intrusion Detection System Using an Improved Hybrid Deep Learning Model for Binary and Multi-Class Classification in IoT Networks. Technologies. 2025;13(3):102. doi: 10.3390/technologies13030102

[30] Nie F, Liu G, Liu W, Huang J, Gao B. IoT-AMLHP: Aligned multimodal learning of header-payload representations for resource-efficient malicious IoT traffic classification. Ad Hoc Networks. 2025;178:103916. doi: 10.1016/j.adhoc.2025.103916

[31] Ahmed U, Jiangbin Z, Almogren A, Khan S, Sadiq MT, Altameem A, et al. Explainable AI-based innovative hybrid ensemble model for intrusion detection. J Cloud Comp. 2024;13(1). doi: 10.1186/s13677-024-00712-x

[32] Tutuncuoglu BT. Invisible failures: how AI uncovers hidden server configuration risks before they break your infrastructure. In: 2023. https://ssrn.com/abstract=5251988

[33] Barredo Arrieta A, Díaz-Rodríguez N, Del Ser J, Bennetot A, Tabik S, Barbado A, et al. Explainable Artificial Intelligence (XAI): Concepts, taxonomies, opportunities and challenges toward responsible AI. Information Fusion. 2020;58:82–115. doi: 10.1016/j.inffus.2019.12.012

[34] Iot Device Network Logs. https://www.kaggle.com/datasets/speedwall10/iot-device-network-logs

[35] UNSW_NB15. https://www.kaggle.com/datasets/mrwellsdavid/unsw-nb15

